# Anharmonic Vibrational States of Double-Well Potentials in the Solid State from DFT Calculations

**DOI:** 10.1021/acs.jctc.4c01394

**Published:** 2025-03-10

**Authors:** Davide Mitoli, Maria Petrov, Jefferson Maul, William B. Stoll, Michael T. Ruggiero, Alessandro Erba

**Affiliations:** † Dipartimento di Chimica, 9314 Università di Torino, via Giuria 5, 10125 Torino, Italy; ‡ Department of Chemistry, 6927 University of Rochester, Rochester, New York 14627, United States

## Abstract

We introduce a general approach for the simulation of quantum vibrational states of (symmetric and asymmetric) double-well potentials in molecules and materials for thermodynamic and spectroscopic applications. The method involves solving the nuclear Schrödinger equation associated with a one-mode potential of the type *V*(*Q*) = *aQ* ^2^ + *bQ* ^3^ + *cQ* ^4^ (with *a* < 0 and *c* > 0) and thus explicitly includes nuclear quantum effects. The potential, *V*(*Q*), is obtained from density functional theory (DFT) calculations performed at displaced nuclear configurations along the selected normal mode, *Q*. The strategy has been implemented into the Crystal electronic structure package and allows for (i) the use of many density functional approximations, including hybrid ones, and (ii) integration with a quasi-harmonic module. The method is applied to the spectroscopic characterization of soft lattice modes in two phases of the molecular crystal of thiourea: a low-temperature ferroelectric phase and a high-temperature paraelectric phase. Signature peaks associated with structural changes between the two phases are found in the terahertz region of the electromagnetic spectrum, which exhibit strong anharmonic character in their thermal evolution, as measured by temperature-dependent terahertz time-domain spectroscopy.

## Graphical abstract

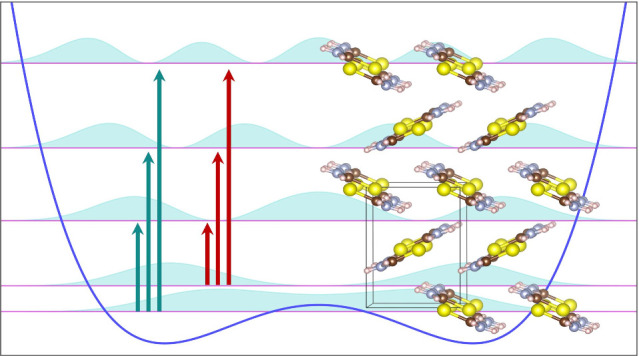

An explicit treatment of nuclear degrees of freedom through statistical mechanics is key to a reliable description of finite temperature properties of materials: stability, structure, phase transitions, transport, and so on. With first-principles simulations based on the Born–Oppenheimer (BO) approximation, lattice dynamics is routinely described within the harmonic approximation (HA), which is valid only for weakly anharmonic systems and for restricted temperature ranges. Moreover, many properties of materials are intrinsically anharmonic and thus can not be captured by the HA (e.g., lattice thermal conductivity, phonon lifetimes, thermal expansion, pyroelectricity).^1–12^ For a given BO potential (potential energy surface, PES), exact anharmonic free energies can be calculated from imaginary time path integral simulations.^13^ However, their prohibitive computational cost has so far prevented their popularity in favor of other, more efficient, approximate approaches, among which the self-consistent harmonic approximation (SCHA) and self-consistent phonon (SCP) theory have become the standard in solid state physics.^14–24^ Within such approaches, anharmonicity is described through an effective temperature-dependent harmonic Hamiltonian with the assumption of Gaussian atomic fluctuations (see Figure 1 for a schematic graphical representation).^17^

**Figure 1:**
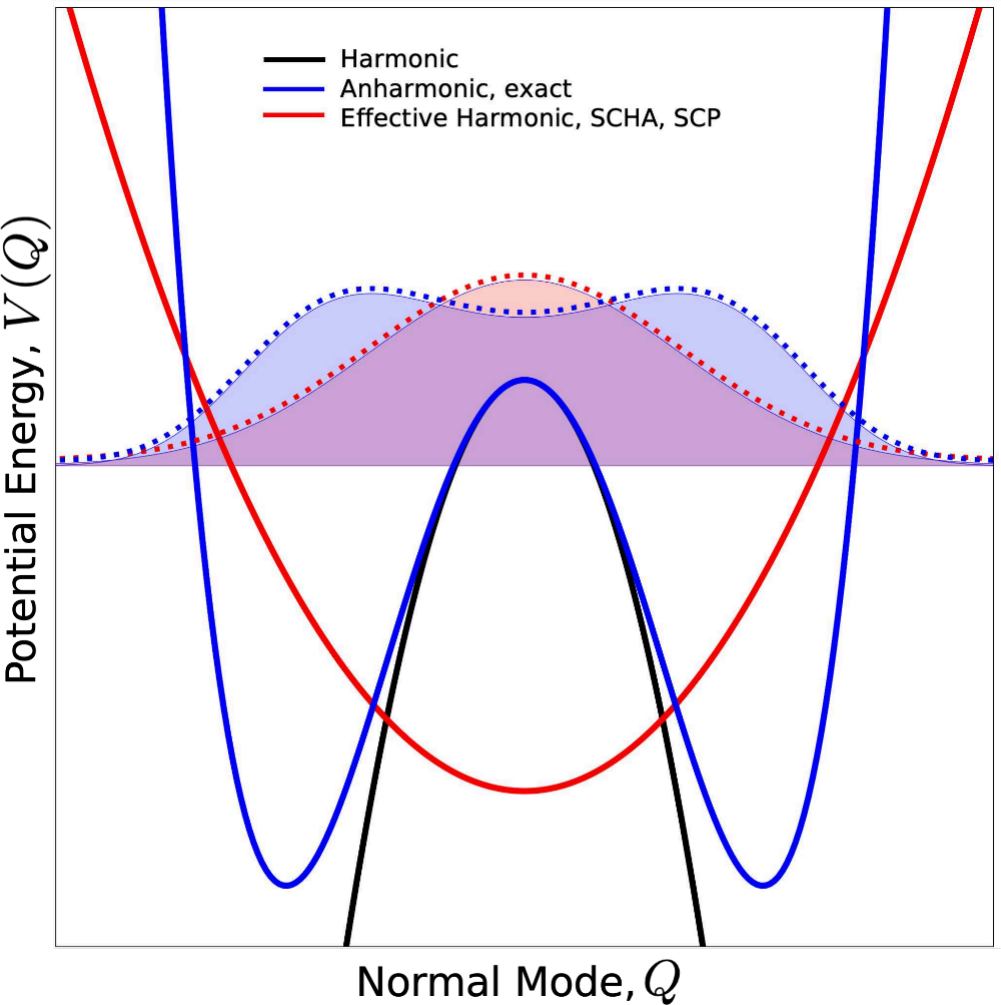
Schematic representation of a strongly anharmonic, symmetric, double-well potential (blue solid line), along with an optimized effective harmonic potential that approximates it within the SCHA and SCP theories (red solid line). The corresponding lowest-energy eigenstates are shown with dashed blue and red lines, respectively. The actual harmonic term of the true potential is shown with a black solid line.

Among strongly anharmonic potentials, the double-well potential (DWP) is ubiquitous in solid state physics as it plays a central role in structural phase transitions, ferroelectricity, proton-transfers, and in general in finite-temperature structure and stability.^22,25–36^ The Gaussian approximation of SCHA and SCP breaks down in this case as quantum tunneling in multiminima energy landscapes yields wave functions that significantly depart from a Gaussian behavior, and instead often exhibit multipeak shapes;^37^ an approach to partially correct for this limitation within the SCHA (namely, nonlinear SCHA) has very recently been formulated.^37^ Figure 1 reports a schematic representation of a strongly anharmonic DWP and of its associated lowest-energy eigenstate (blue lines), and a comparison with an effective harmonic potential and corresponding eigenstate that approximates it within the SCHA and SCP approaches (red lines). In this Letter, we present a general method to obtain both energy levels and wave functions of vibrational states of symmetric and asymmetric DWPs in solids. Solutions of the nuclear Schrödinger equation are found, associated with an anharmonic PES derived from density functional theory (DFT) calculations. The method allows for fully including the true anharmonic character of vibrational potentials to compute anharmonic wave functions, leading to an accurate description of properties that depend explicitly on anharmonic states, such as spectroscopic transitions, thermal displacement parameters, pyroelectricity, and so on.

The proposed strategy has been implemented in a developer’s version of the Crystal23 electronic structure package^38^ and represents an extension of the anharmonic module thereof for: (i) calculation of cubic and quartic interatomic force constants;^39,40^ (ii) nonperturbative many-body solution of the nuclear Schrödinger equation via the vibrational self-consistent field (VSCF) and vibrational configuration interaction (VCI) methods;^41–43^ (iii) infrared and Raman anharmonic vibrational spectroscopy.^44,45^

The method starts with the calculation of phonon frequencies ω_*i*_ and normal modes *Q*_*i*_ (with *i* = 1,···,3 *N*, where *N* represents the number of atoms per cell) from the HA. Normal mode coordinates associated with DWPs are identified from imaginary frequencies. We work within a quartic representation of the DWP as follows:

1 $$V=aQ^{2}+bQ^{3}+cQ^{4}$$

with *a* < 0 and *c* > 0. The coefficient *b* of the cubic term determines the skewness of the potential (symmetric for *b* = 0, asymmetric for *b* ≠ 0). The accurate numerical evaluation of the *a*, *b* and *c* parameters of the DWP of eq 1 requires some care, especially when the wells are shallow. For instance, they can be computed in terms of second-, third- and fourth-order energy derivatives with respect to *Q* via finite-difference approaches, see eq (S1) in the Supporting Information. Three different finite-difference approaches have been implemented in Crystal for evaluation of cubic and quartic interatomic force constants, some based only on the energy and others based on both the energy and the (analytical) forces.^39^ The most efficient approach belongs to the latter class, and requires calculations at just two displaced nuclear configurations at ± δ along *Q*.^39,46^ With this approach, we find the quartic coefficient *c* to significantly depend on the step δ. In particular, a larger step than usually set for less anharmonic potentials provides better results (δ = 1.5 relative to the default value of 0.9, in units of classical amplitude). An alternative approach, based on a denser and more extended sampling of the PES, consists in computing the energy values at several displaced nuclear configurations along the normal mode and best-fit them to a polynomial representation of *V*(*Q*) as in eq 1 to obtain the *a*, *b* and *c* parameters. Here, the most critical factor is the explored *Q* range. In general this approach provides more accurate results at a higher cost. We analyze the numerical performance of these approaches in the Supporting Information.

We consider a one-mode nuclear Hamiltonian:

2 $$H=-\frac{1}{2}\frac{\partial^{2}}{\partial Q^{2}}+V\left(Q\right)$$

where *V*(*Q*) is the DWP of eq 1, and look for its solutions *HΨ*_*s*_ = *E*_*s*_ Ψ_*s*_ (with *s* being a state index). We express the anharmonic wave functions Ψ_*s*_ in an appropriately selected (see below) harmonic basis:

3 $$\Psi_{s}\left(Q\right)=\overset{M}{\underset{\mu =1}{\sum }}c_{\mu ,s}\psi_{\mu }\left(Q;\alpha \right)$$

where ψ_μ_(*Q*;α) are the solutions of a quantum harmonic oscillator with potential $\frac{1}{2}\alpha^{4}Q^{2}$ :

$$\psi_{\mu }\left(Q;\alpha \right)={\left(\frac{\alpha }{\sqrt{\pi }2^{\mu }\mu !}\right)}^{\frac{1}{2}}H_{\mu }\left(\xi \right)e^{-\frac{\xi^{2}}{2}}$$ 4

with ξ = *Qα* and *H*_μ_ being the μ-th order Hermite polynomial. To determine the optimal harmonic basis (i.e., optimal value of the parameter α) for the description of the DWP of eq 1, we follow the strategy first suggested by Balsa et al. and set $\alpha^{2}=\sqrt{2\left|a\right|}$ .^47^ Other choices for the value of α (i.e., the scale of the harmonic basis) are possible. Upon linearization as in eq 3, the nuclear Schrödinger equation can be expressed in matrix form, **HC** = **EC**, and its solutions obtained by diagonalization of the symmetric Hamiltonian matrix **H**, whose nonvanishing elements have closed analytical expressions given below:

$$\begin{array}{lll} \left\langle \mu \right|H\left|\mu \right\rangle & \equiv & \left\langle \mu \right|T+aQ^{2}+cQ^{4}\left|\mu \right\rangle \\ & = & \left(\frac{\alpha^{4}+2a}{2\alpha^{2}}\right)\left(\mu +\frac{1}{2}\right)+\frac{3c}{4\alpha^{4}}\left(2\mu^{2}+2\mu +1\right)\\ \left\langle \mu \right|H\left|\mu +1\right\rangle & \equiv & \left\langle \mu \right|bQ^{3}\left|\mu +1\right\rangle \\ & = & \frac{3b}{2}\sqrt{\frac{{\left(\mu +1\right)}^{3}}{2\alpha^{6}}}\\ \left\langle \mu \right|H\left|\mu +2\right\rangle & \equiv & \left\langle \mu \right|T+aQ^{2}+cQ^{4}\left|\mu +2\right\rangle \\ & = & \sqrt{\left(\mu +1\right)\left(\mu +2\right)}\\ & \times & \left[-\frac{\alpha^{2}}{4}+\frac{a}{2\alpha^{2}}+\frac{c}{2\alpha^{4}}\left(2\mu +3\right)\right]\\ \left\langle \mu \right|H\left|\mu +3\right\rangle & \equiv & \left\langle \mu \right|bQ^{3}\left|\mu +3\right\rangle \\ & = & b\sqrt{\frac{\left(\mu +1\right)\left(\mu +2\right)\left(\mu +3\right)}{8\alpha^{6}}}\\ \left\langle \mu \right|H\left|\mu +4\right\rangle & \equiv & \left\langle \mu \right|cQ^{4}\left|\mu +4\right\rangle \\ & = & \frac{c}{4\alpha^{4}}\sqrt{\left(\mu +1\right)\left(\mu +2\right)\left(\mu +3\right)\left(\mu +4\right)} \end{array}$$ 5

where *T* represents the kinetic term of the nuclear Hamiltonian. It is crucial to check for convergence of the obtained anharmonic states with respect to the size of the adopted harmonic basis–i.e. with respect to the number *M* of basis functions used in eq 3 – both in terms of energies *E*_*s*_ and wave functions Ψ_*s*_. We address this aspect in Figure 2 for symmetric and asymmetric DWPs. In both cases, we analyze the 20 lowest lying anharmonic states. Panels A) and C) report a graphical representation of the fully converged (*M* → ∞, i.e. M = 500 in this case) exact solutions in terms of vibrational energy levels and square-modulus of the corresponding wave functions (the actual wave functions are shown in Figure S3 of the Supporting Information). Panels B) and D) show the convergence of the anharmonic energy levels *E*_*s*_ with respect to *M*. The reported quantity, for each state *s*, is log_10_[|*E*_*s*_(*M*) – *E*_*s*_(*M* → ∞)|], which is given on a color scale. The following can be observed: (i) the computed anharmonic states regularly converge as *M* increases (for both potentials, *M* ≥ 60 provides stable results); (ii) the lowest energy states converge with *M* faster than the higher energy ones; (iii) the convergence with *M* is faster for symmetric rather than asymmetric potentials.

**Figure 2:**
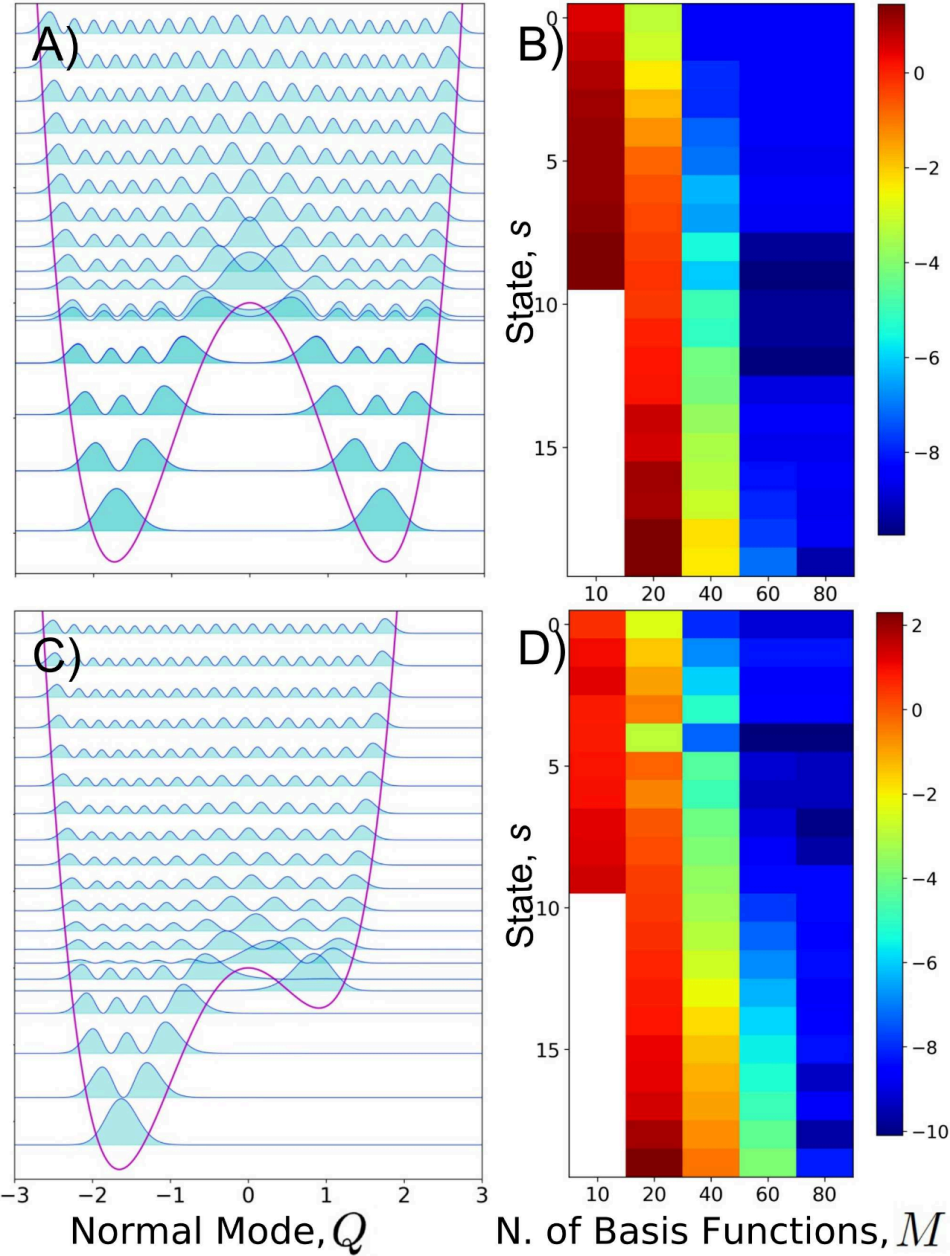
A) The 20 lowest lying anharmonic states (vibrational energy levels and square-modulus of the corresponding wave functions) of a symmetric DWP with *a* = −30 and *c* = 5. B) Convergence of the 20 lowest lying anharmonic states of the DWP represented in panel A) as a function of the number of basis functions used, *M*; for each state *s*, the reported quantity is log_10_[|*E*_*s*_(*M*) – *E*_*s*_(*M* → ∞)|]. C) Same as in A) but for an asymmetric DWP with *a* = −30, *b* = 10 and *c* = 10. D) Same as in B) but for the asymmetric potential of panel C). Plots in panels A) and C) are produced with the CRYSTALpytools Python interface to Crystal.^48^

We apply the methodology described above to the molecular crystal of thiourea to document its effectiveness. Thiourea has a rich polymorphism as a function of temperature, pressure, applied electric field, and is the second oldest ferroelectric to exhibit an incommensurate phase, between 169 and 202 K.^49–51^ Below 169 K, thiourea crystallizes in a ferroelectric orthorhombic P2_1ma_ lattice (low-temperature phase). Above 202 K, a paraelectric orthorhombic phase is stabilized with a lattice of *Pnma* space group symmetry (high-temperature phase). These two phases are very similar, with four molecules per unit cell and a structure consisting of staggered ribbons along the **b** axis. The molecules are polar. In the high-temperature, paraelectric, phase, the dipole moments of two pairs of adjacent chains cancel out due to symmetry, resulting in no net dipole moment. On the other hand in the low-temperature, ferroelectric, phase, the molecules slightly tilt and shift in the **ac** plane (compared to the high-temperature phase), resulting in a nonvanishing net polarization.^52^ Figure 3 A) and B) reports a view down the **b** axis of the crystal structure of these two phases of thiourea. The ferroelectric and incommensurate phase transitions only involve molecular motions in the **ac** plane. Soft lattice vibrations associated with such motions were probed by vibrational spectroscopies (infrared reflection and Raman scattering) in the 1970s and were found to produce peaks in the terahertz region of the spectrum.^53,54^ In particular, their temperature dependence was analyzed in the temperature region 20–300 K, which showed remarkable anharmonic features, including the pronounced broadening of the lowest active infrared peak. Here, we find this peak to be associated with a DWP in the high-temperature paraelectric phase, which explains the observed broadening (see below).

**Figure 3:**
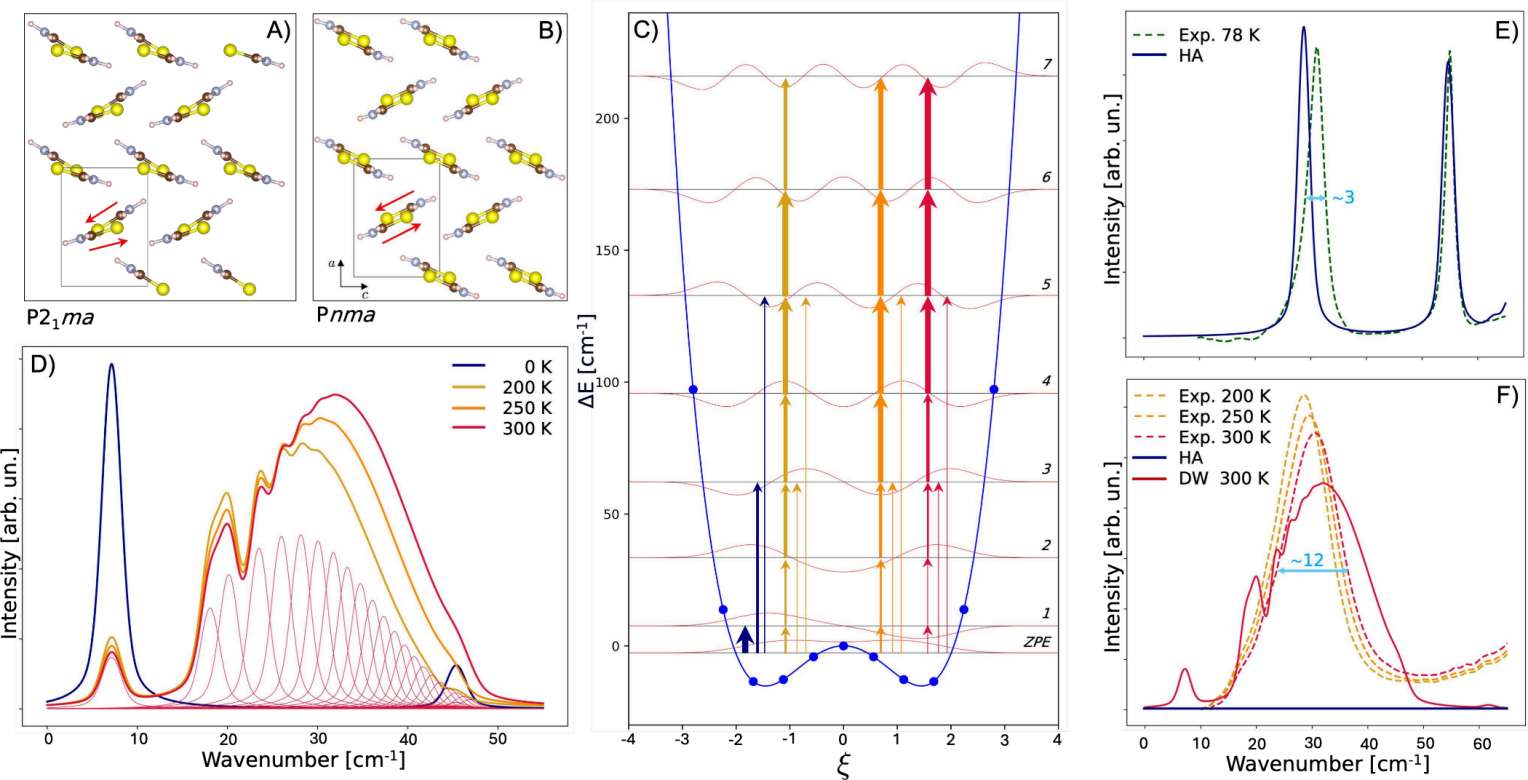
A) and B) Crystal structure of the low-temperature ferroelectric P2_1_ *ma* and high-temperature paraelectric P*nma* orthorhombic phase of thiourea, respectively (viewed down the **b** axis). C) The DWP of the P*nma* phase (blue circles and line) as derived from DFT calculations, along with the solutions–energy levels and wave functions–to its associated nuclear Schrödinger equation. The arrows mark active infrared transitions with intensities proportional to their thickness (dark blue, 0 K, yellow, 200 K, orange, 250 K, red, 300 K). D) Simulated infrared spectrum associated with the DWP of panel C) at four different temperatures. Single transitions contributing to the overall spectrum at 300 K are reported as thin red lines. E) Infrared spectrum of the low-temperature ferroelectric phase in the 0–65 cm^–1^ spectral range (dashed line for experiment at 78 K, blue solid line for the HA simulation). F) Infrared spectrum of the high-temperature paraelectric phase in the 0–65 cm^–1^ spectral range (dashed lines for experiment at 200, 250, and 300 K, blue solid line for the HA simulation, red solid line for the anharmonic simulation). All simulated spectra are scaled by 0.7 to match the experimental peak positions.

Experimental terahertz time-domain spectroscopy measurements were performed on commercially acquired microcrystalline powders of thiourea (Sigma-Aldrich, > 99%). The sample was used as-received, mixed with polytetrafluoroethylene (PTFE) to a 1% w/w concentration, homogenized with a mortar and pestle, and pressed into 13 mm diameter free-standing pellets with a width of ca. 3 mm. A pellet of pure PTFE with the same dimensions was created and served as a spectral reference. The samples were placed in a liquid nitrogen cryostat (Lakeshore Cryotronics), which permitted acquisition at temperatures from 78 to 300 K. The terahertz spectra were acquired using a commercial terahertz spectrometer (Toptica Photonics), which consisted of a pair of fiber-coupled emitter and receiver modules, and a corresponding free-space optical setup for collimating and focusing the terahertz radiation on the sample and receiver. The recorded terahertz time-domain waveforms were a result of 20,000 individual averaged data sets, which were subsequently Fourier transformed to yield frequency-domain power spectra. An absorption spectrum was generating by taking the ratio of a sample and blank power spectrum, and the spectra shown in Figure 3 E) and F) (dashed lines) are a result of averaging four individual absorption spectra in the frequency domain. The experimental spectra were acquired at select temperatures: 78 K for the ferroelectric P2_1_ *ma* phase and 200, 250, and 300 K for the paraelectric P*nma* phase. Two narrow peaks are present at low temperature in the ferroelectric phase at about 30 and 55 cm^–1^. The second peak disappears in the high-temperature paraelectric phase while the first one broadens very significantly; as temperature increases from 200 to 300 K, the first peak slightly shifts to higher wavenumbers and progressively broadens.

We perform DFT calculations with the Crystal electronic structure package^38,55^ on the low- and high-temperature phases of thiourea with the hybrid B3LYP exchange-correlation functional,^56^ as corrected for missing dispersive interactions following Grimme’s -D3 approach.^57^ A triple-ζ quality basis set specifically optimized for solid state calculations is used.^58^ Full structural relaxation is achieved through a geometry optimization process, followed by the evaluation of harmonic frequencies and normal modes of vibration. All computed harmonic frequencies are positive in the low-temperature P2_1_ *ma* structure, while the high-temperature P*nma* structure exhibits one negative eigenvalue of the mass-weighted Hessian, which could be indicative of a DWP. We determine the coefficients of the DWP in the paraelectric phase of thiourea by computing the DFT energy at 11 displaced nuclear configurations along the corresponding normal mode and by fitting them to the potential in eq 1, see blue circles and line in Figure 3 C). The solutions–energy levels and wave functions–of the associated nuclear Schrödinger equation are also shown in Figure 3 C), as obtained following the approach outlined above, with M = 150, which ensures full convergence of all spectroscopically relevant states.

Key to a correct description of the spectroscopic fingerprint of a DWP is the description of anharmonic “hot bands” (i.e., spectral features associated with transitions between two vibrationally excited states). Infrared (IR) intensities associated with a transition between an initial state Ψ_*i*_, with energy *E*_*i*_, and a final state Ψ_*f*_ can be modeled as

6 $$I_{i\to f}^{\mathrm{IR}}\left(T\right)=I_{i\to f}^{\mathrm{IR},0}\times p_{i}\left(T\right)$$

By expanding the dipole moment **μ** in a Taylor series with respect to *Q* and truncating to first-order (i.e., neglecting electrical anharmonicity), the first term on the right-hand side of eq 6 reads:

7 $$I_{i\to f}^{\mathrm{IR},0}=\underset{a=x,y,z}{\sum }{\left(\frac{\partial \mu_{a}}{\partial Q}\right)}^{2}\left\langle i\right|Q|f\rangle^{2}$$

where the derivatives constitute the Born tensor that we compute either numerically through a Berry phase approach or analytically through a coupled-perturbed (CP) approach.^59,60^ The last term in eq 6 represents the statistical probability of the initial state to be thermally populated:

$$p_{i}\left(T\right)=\frac{1}{Z}e^{-\frac{E_{i}}{k_{B}T}}\;\mathrm{with}\;Z=\overset{\mathrm{all}}{\underset{s}{\sum }}e^{-\frac{E_{s}}{k_{B}T}}$$ 8

The main active IR transitions associated with the DWP of thiourea are schematically shown in Figure 3 C) at four selected temperatures (0, 200, 250, and 300 K) by arrows with different thickness (proportional to the corresponding intensity). At 0 K only transitions starting from the fundamental state would be described (dark blue arrows). At higher temperatures, a multitude of “hot band” transitions occur, which determine the overall broadening of the DW spectral feature in the infrared spectrum. This is shown in Figure 3 D) where we report the simulated IR spectrum of the paraelectric phase of thiourea at four temperatures. Neglecting thermal effects (i.e., no “hot bands” at 0 K) results in a narrow peak at about 7 cm^–1^. Simulations at finite temperatures corresponding to the stability domain of the paraelectric phase, show several distinctive features: (i) a broad spectral feature centered at about 30 cm^–1^; (ii) a slight shift of the peak to higher wavenumbers as a function of temperature; (iii) a slight increase in the broadening as a function of temperature. For the 300 K case, red lines, we also report the individual transitions contributing to the total profile to highlight the role of the multiple “hot bands” in determining the shape of the spectral feature of a DWP.

We compare the simulated spectra to the experimental ones in the 0–65 cm^–1^ spectral range in Figure 3 E) and F). In particular, we show the simulated IR spectrum for the low-temperature ferroelectric phase as obtained from the HA (blue solid line) in panel E). The HA completely breaks down in the high-temperature paraelectric phase, where it predicts no peaks in the whole 0–65 cm^–1^ range, see blue solid line in panel F). The simulated spectrum from the anharmonic approach of the DWP discussed above is shown as a solid red line, as modeled at 300 K, which matches rather remarkably with the experiment.

In conclusion, we have presented a theoretical method and a computational protocol to effectively describe vibrational states associated with strongly anharmonic, double-well, potentials within DFT calculations. The effectiveness of the presented approach has been discussed on a DWP found in the high-temperature paraelectric phase of the thiourea molecular crystal. Work is currently in progress to extend this approach to couplings of the DWP with other modes, and to treat electrical anharmonicity.

## Supplementary Material



## Notes

Details for the numerical evaluation of the coefficients of the DWP. Effect of higher than quartic terms in the description of the DWP. Graphical representation of the wave functions of the toy model symmetric and asymmetric DWPs. Effect of volume expansion on the DWP (PDF)

## References

[1] Garg J., Bonini N., Kozinsky B., Marzari N. (2011). Role of disorder and
anharmonicity in the thermal conductivity of silicon-germanium alloys:
A first-principles study. Phys. Rev. Lett..

[2] Hellman O., Abrikosov I. A., Simak S. I. (2011). Lattice dynamics of anharmonic solids
from first principles. Phys. Rev. B.

[3] Errea I., Calandra M., Mauri F. (2013). First-principles
theory of anharmonicity
and the inverse isotope effect in superconducting palladium-hydride
compounds. Phys. Rev. Lett..

[4] Bansal D., Hong J., Li C. W., May A. F., Porter W., Hu M. Y., Abernathy D. L., Delaire O. (2016). Phonon anharmonicity
and negative thermal expansion in SnSe. Phys.
Rev. B.

[5] Linnera J., Erba A., Karttunen A. J. (2019). Negative
thermal expansion of Cu_2_O studied by quasi-harmonic approximation
and cubic force-constant
method. J. Chem. Phys..

[6] Zhou F., Nielson W., Xia Y., Ozoliņš V. (2014). Lattice anharmonicity
and thermal conductivity from compressive sensing of first-principles
calculations. Phys. Rev. Lett..

[7] Tadano T., Gohda Y., Tsuneyuki S. (2014). Anharmonic
force constants extracted
from first-principles molecular dynamics: applications to heat transfer
simulations. J. Phys.: Condens. Matter.

[8] Rossi M., Gasparotto P., Ceriotti M. (2016). Anharmonic and quantum fluctuations
in molecular crystals: A first-principles study of the stability of
paracetamol. Phys. Rev. Lett..

[9] Eklund K., Karttunen A. J. (2023). Pyroelectric
Effect in Tetragonal Ferroelectrics BaTiO3
and KNbO3 Studied with Density Functional Theory. J. Phys. Chem. C.

[10] Liu J., Pantelides S. T. (2018). Mechanisms of pyroelectricity in
three-and two-dimensional
materials. Phys. Rev. Lett..

[11] Ruggiero M. T., Zeitler J., Erba A. (2017). Intermolecular
Anharmonicity in Molecular
Crystals: Interplay between Experimental Low-Frequency Dynamics and
Quantum Quasi-Harmonic Simulations of Solid Purine. Chem. Commun..

[12] Juneja N., Hastings J. L., Stoll W. B., Brennessel W. W., Zarrella S., Sornberger P., Catalano L., Korter T. M., Ruggiero M. T. (2024). Fundamentally intertwined: anharmonic intermolecular
interactions dictate both thermal expansion and terahertz lattice
dynamics in molecular crystals. Chem. Commun..

[13] Kapil V., Engel E., Rossi M., Ceriotti M. (2019). Assessment of approximate
methods for anharmonic free energies. J. Chem.
Theory Comput..

[14] Hooton D. LI. (1955). A new
treatment of anharmonicity in lattice thermodynamics: I.. London Edinburgh Philos. Mag. & J. Sci..

[15] Werthamer N. (1970). Self-consistent
phonon formulation of anharmonic lattice dynamics. Phys. Rev. B.

[16] Souvatzis P., Eriksson O., Katsnelson M. I., Rudin S. P. (2008). Entropy Driven Stabilization
of Energetically Unstable Crystal Structures Explained from First
Principles Theory. Phys. Rev. Lett..

[17] Errea I., Calandra M., Mauri F. (2014). Anharmonic
free energies and phonon
dispersions from the stochastic self-consistent harmonic approximation:
Application to platinum and palladium hydrides. Phys. Rev. B.

[18] Monacelli L., Bianco R., Cherubini M., Calandra M., Errea I., Mauri F. (2021). The stochastic self-consistent
harmonic approximation: calculating
vibrational properties of materials with full quantum and anharmonic
effects. J. Phys.: Condens. Matter.

[19] Monacelli L., Mauri F. (2021). Time-dependent self-consistent
harmonic approximation: Anharmonic
nuclear quantum dynamics and time correlation functions. Phys. Rev. B.

[20] Siciliano A., Monacelli L., Caldarelli G., Mauri F. (2023). Wigner Gaussian dynamics:
Simulating the anharmonic and quantum ionic motion. Phys. Rev. B.

[21] Tadano T., Tsuneyuki S. (2015). Self-consistent phonon calculations of lattice dynamical
properties in cubic SrTiO 3 with first-principles anharmonic force
constants. Phys. Rev. B.

[22] Zacharias M., Volonakis G., Giustino F., Even J. (2023). Anharmonic
lattice
dynamics via the special displacement method. Phys. Rev. B.

[23] Schiltz C., Rappoport D., Mandelshtam V. A. (2023). Implementation of the self-consistent
phonons method with ab initio potentials (AI-SCP). J. Chem. Phys..

[24] Monacelli, L. Simulating anharmonic crystals: Lights and shadows of first-principles approaches. arXiv preprint arXiv:2407.03090 2024.

[25] Tadano T., Tsuneyuki S. (2019). Ab initio
prediction of structural phase-transition
temperature of SrTiO3 from finite-temperature phonon calculation. J. Ceram. Soc. Jpn..

[26] Hoffmann M., Fengler F. P., Herzig M., Mittmann T., Max B., Schroeder U., Negrea R., Lucian P., Slesazeck S., Mikolajick T. (2019). Unveiling the double-well energy landscape in a ferroelectric
layer. Nature.

[27] Konwent H. (1986). On the Application
of a New Type Double-Well Potential in the Theory of Ferroelectric. Phase Transitions. Phys. Status Solidi B.

[28] Choudhury R. R., Chitra R., Ramanadham M. (2003). The role of
the double-well potential
seen by the amino group in the ferroelectric phase transition in triglycine
sulfate. J. Phys.: Condens. Matter.

[29] Wang C., Arago C., Garcia J., Gonzalo J. (2002). Quantum tunneling versus
zero-point energy in double-well potential model for ferrroelectric
phase transitions. Phys. A: Stat. Mech. Appl..

[30] Fillaux F., Nicolai B., Baron M., Lautie A., Tomkinson J., Kearley G. (1998). A new view of the quantum
dynamics for proton transfer
along hydrogen bonds: Vibrational spectroscopy with neutrons. Ber. Bunsenges. Phys. Chem..

[31] Fillaux F. (2002). The impact
of vibrational spectroscopy with neutrons on our view of quantum dynamics
in hydrogen bonds and proton transfer. J. Mol.
Struct..

[32] Xu Z.-H., Meuwly M. (2017). Vibrational
spectroscopy and proton transfer dynamics
in protonated oxalate. J. Phys. Chem. A.

[33] Krasilnikov P. (2014). Two-dimensional
model of a double-well potential: Proton transfer upon hydrogen bond
deformation. Biophys..

[34] Eckold G., Grimm H., Stein-Arsic M. (1992). Proton disorder
and phase transition
in KHCO3. Physica B: Condensed Matter.

[35] Cailleau H., Baudour J.-L., Meinnel J., Dworkin A., Moussa F., Zeyen C. M. (1980). Double-well potentials
and structural phase transitions
in polyphenyls. Faraday Discuss..

[36] Goryainov S. (2012). A model of
phase transitions in double-well Morse potential: Application to hydrogen
bond. Physica B: Condensed Matter.

[37] Siciliano A., Monacelli L., Mauri F. (2024). Beyond Gaussian fluctuations of quantum
anharmonic nuclei: The case of rotational degrees of freedom. Phys. Rev. B.

[38] Erba A., Desmarais J. K., Casassa S., Civalleri B., Doná L., Bush I. J., Searle B., Maschio L., Edith-Daga L., Cossard A., Ribaldone C., Ascrizzi E., Marana N. L., Flament J.-P., Kirtman B. (2023). CRYSTAL23:
A Program for Computational Solid State Physics and Chemistry. J. Chem. Theory Comput.h.

[39] Erba A., Maul J., Ferrabone M., Carbonniére P., Rérat M., Dovesi R. (2019). Anharmonic Vibrational
States of
Solids from DFT Calculations. Part I: Description of the Potential
Energy Surface. J. Chem. Theory Comput..

[40] Mitoli D., Maul J., Erba A. (2023). Anharmonic
Terms of the Potential
Energy Surface: A Group Theoretical Approach. Cryst. Growth Des..

[41] Erba A., Maul J., Ferrabone M., Dovesi R., Rérat M., Carbonnière P. (2019). Anharmonic Vibrational States of Solids from DFT Calculations.
Part II: Implementation of the VSCF and VCI Methods. J. Chem. Theory Comput.h.

[42] Maul J., Spoto G., Mino L., Erba A. (2019). Elucidating the structure
and dynamics of CO ad-layers on MgO surfaces. Phys. Chem. Chem. Phys..

[43] Schireman R. G., Maul J., Erba A., Ruggiero M. T. (2022). Anharmonic Coupling
of Stretching Vibrations in Ice: A Periodic VSCF and VCI Description. J. Chem. Theory Comput..

[44] Carbonnière P., Erba A., Richter F., Dovesi R., Rérat M. (2020). Calculation
of anharmonic IR and Raman intensities for periodic systems from DFT
calculations: Implementation and validation. J. Chem. Theory Comput..

[45] Mitoli D., Maul J., Erba A. (2024). First-Principles Anharmonic
Infrared
and Raman Vibrational Spectra of Materials: Fermi Resonance in Dry
Ice. J. Phys. Chem. Lett..

[46] Lin C. Y., Gilbert A. T., Gill P. M. (2008). Calculating
molecular vibrational
spectra beyond the harmonic approximation. Theor.
Chem. Acc..

[47] Balsa R., Plo M., Esteve J., Pacheco A. (1983). Simple procedure to compute accurate
energy levels of a double-well anharmonic oscillator. Phys. Rev. D.

[48] Camino B., Zhou H., Ascrizzi E., Boccuni A., Bodo F., Cossard A., Mitoli D., Ferrari A. M., Erba A., Harrison N. M. (2023). CRYSTALpytools:
a Python Infrastructure for the CRYSTAL
Code. Comput. Phys. Commun..

[49] McKenzie D., Dryden J. (1973). Dielectric properties and ferroelectric
transitions
of thiourea. J. Phys. C: Solid State Phys..

[50] Klimowski J., Wanarski W., Ożgo D. (1976). Effect of
hydrostatic pressure on
the phase transition temperatures and spontaneous polarization of
thiourea monocrystals. Phys. Status Solidi A.

[51] Dove M. T., Lynden-bell R. M. (1986). A model
of the paraelectric phase of thiourea. Phylos.
Mag. B.

[52] Elcombe M. M., Taylor J. (1968). A neutron diffraction determination of the crystal
structures of thiourea and deuterated thiourea above and below the
ferroelectric transition. Acta Crystallogr.
A.

[53] Siapkas D. (1980). Soft mode
in the disordered-incommensurate-commensurate phase transitions of
thiourea. Ferroelectrics.

[54] Petzelt J. (1981). Dielectric
and light scattering spectroscopy of incommensurate phases in crystals. Ph. Transit..

[55] Erba A., Baima J., Bush I., Orlando R., Dovesi R. (2017). Large Scale
Condensed Matter DFT Simulations: Performance and Capabilities of
the Crystal Code. J. Chem. Theory Comput..

[56] Becke A. D. (1993). Density-functional
thermochemistry. III. The role of exact exchange. J. Chem. Phys..

[57] Grimme S., Antony J., Ehrlich S., Krieg H. (2010). A consistent and accurate
ab initio parametrization of density functional dispersion correction
(DFT-D) for the 94 elements H-Pu. J. Chem. Phys..

[58] Vilela
Oliveira D., Laun J., Peintinger M. F., Bredow T. (2019). BSSE-correction scheme for consistent gaussian basis
sets of double-and triple-zeta valence with polarization quality for
solid-state calculations. J. Comput. Chem..

[59] Maschio L., Kirtman B., Orlando R., Rèrat M. (2012). Ab initio
analytical infrared intensities for periodic systems through a coupled
perturbed Hartree-Fock/Kohn-Sham method. J.
Chem. Phys..

[60] Dovesi R., Kirtman B., Maschio L., Maul J., Pascale F., Rérat M. (2019). Calculation
of the infrared intensity of crystalline
systems. A comparison of three strategies based on berry phase, wannier
function, and coupled-perturbed Kohn-Sham methods. J. Phys. Chem. C.

