# Supplementary material for: Anharmonic Vibrational States of Double-Well Potentials in the Solid State from DFT Calculations
Source: J Chem Theory Comput. 2025 Mar 10;21(11):5365–71. doi: 10.1021/acs.jctc.4c01394 (PMC12159970; doi:10.1021/acs.jctc.4c01394)
Supplement: Supplementary file 1 [file ct4c01394_si_001.pdf]

Supporting Information: Anharmonic Vibrational States of Double-Well Potentials in the Solid State from DFT Calculations

Davide Mitoli,† Maria Petrov,† Jefferson Maul,† William B. Stoll,‡ Michael T. Ruggiero,*,‡ and Alessandro Erba*,†

†*Dipartimento di Chimica, Università di Torino, via Giuria 5, 10125 Torino, Italy*

‡*Department of Chemistry, University of Rochester, Rochester, New York 14627, United States*

E-mail: michael.ruggiero@rochester.edu; alessandro.erba@unito.it

1 Numerical Evaluation of the Parameters of the DWP

We consider a 1D double-well potential (DWP) of the type: $V = aQ^2 + bQ^3 + cQ^4$. DFT calculations are performed at the B3LYP-D3/Ahlrichs-pVTZ level on the paraelectric phase of thiourea.

1.1 Finite Difference Approach

Within a finite-difference approach, the a, b, c parameters of the DWP can be expressed in terms of harmonic frequencies and third- and fourth-order energy derivatives as:

$$a = \frac{1}{2}\omega^2, \quad b = \frac{1}{3!}\frac{\partial^3 V}{\partial Q^3}\bigg|_{Q=0}, \quad c = \frac{1}{4!}\frac{\partial^4 V}{\partial Q^4}\bigg|_{Q=0}. \tag{S1}$$

We use the EGH finite-difference approach implemented in CRYSTAL, see scheme 3 in *J. Chem. Theory Comput.*, **15**, 3755-3765 (2019), where only three nuclear configurations are explored (equilibrium and two displaced along Q, at $\pm\delta$).

Table S1: Effect of the step δ on third- and forth-order energy derivatives and first three energy levels of the associated DWP when the PES is sampled with the finite difference EGH scheme 3. All values in cm^{-1}.

Step	ω	$\partial^3 V/\partial Q^3$	$\partial^4 V/\partial Q^4$	E_0	E_1	E_2	Δ_{0-1}	Δ_{0-2}
0.8	i30.03	0.00	137.68	2.18	20.03	53.72	17.85	51.54
1.0	i30.03	0.00	127.17	1.46	18.17	50.74	16.71	49.28
1.5	i30.03	0.00	107.93	-0.08	14.32	44.73	14.40	44.81
2.0	i30.03	0.00	100.00	-0.84	12.5	41.97	13.34	42.81

1.2 Polynomial Fitting of the Energy

A second approach is based on a polynomial fitting of the energy computed at several nuclear configurations obtained by displacing the atoms along the normal mode Q.

Table S2: Effect of the number of explored nuclear configurations along Q on the fitting coefficients. For consistency with Table S1, we report data in terms of second-, third- and fourth-order energy derivatives. The first three energy levels of the associated DWP are also reported. The explored range is [-4, 4] in all three cases, in units of classical amplitude. All values in cm^{-1}.

Points	ω	$\partial^3 V/\partial Q^3$	$\partial^4 V/\partial Q^4$	E_0	E_1	E_2	Δ_{0-1}	Δ_{0-2}
11	i22.04	-0.02	87.47	1.92	17.33	46.31	15.41	44.39
21	i22.28	-0.02	87.79	1.84	17.17	46.15	15.33	44.31
31	i22.22	-0.02	87.74	1.86	17.22	46.20	15.36	44.34

Table S3: Effect of the explored range (in units of classical amplitude) on the fitting coefficients. For consistency with Table S1, we report data in terms of second-, third- and fourth-order energy derivatives. The first three energy levels of the associated DWP are also reported. All values in cm^{-1}.

Range	Points	ω	$\partial^3 V/\partial Q^3$	$\partial^4 V/\partial Q^4$	E_0	E_1	E_2	Δ_{0-1}	Δ_{0-2}
$[-2.0, 2.0]$	11	i26.10	0.02	93.60	0.52	14.82	43.98	14.30	43.46
$[-2.8, 2.8]$	11	i25.81	0.01	92.54	0.57	14.86	43.92	14.29	43.35
$[-6.0, 6.0]$	31	i8.65	-0.72	78.64	6.76	27.51	58.19	20.75	51.43

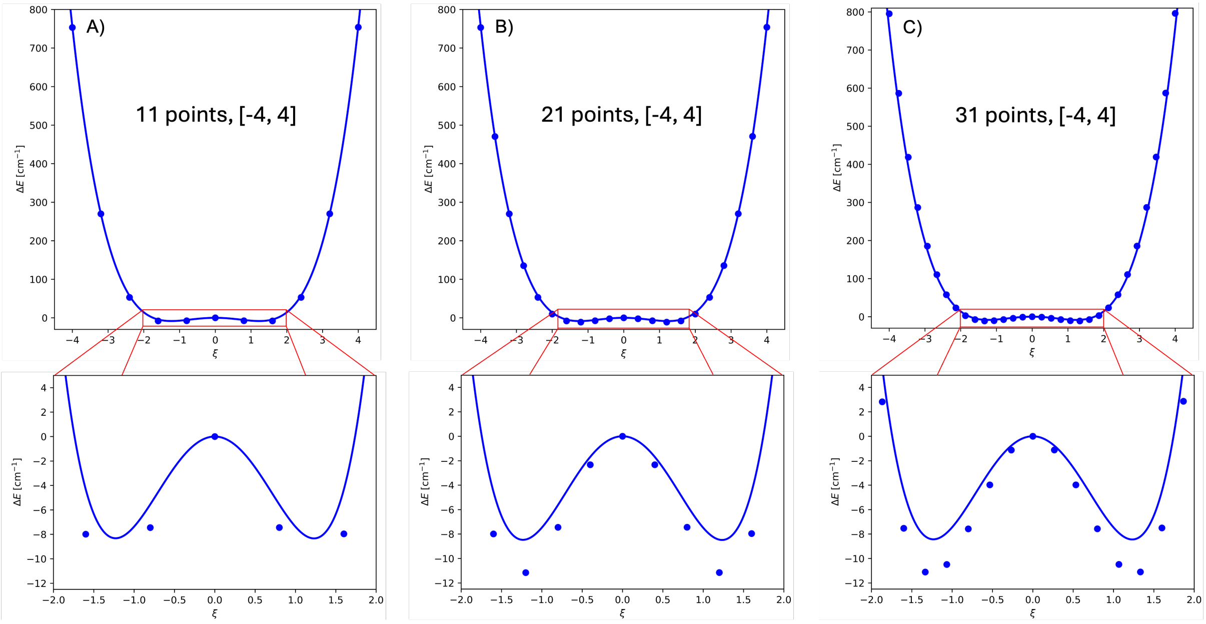

Figure S1: Energy scan and fitting of the DWP of the Pnma phase of thiourea for the range [-4, 4] with a different number of energy points: 11 (A), 21 (B) and 31 (C). Zoomed regions in the range [-2, 2] are displayed to compare the goodness of fit.

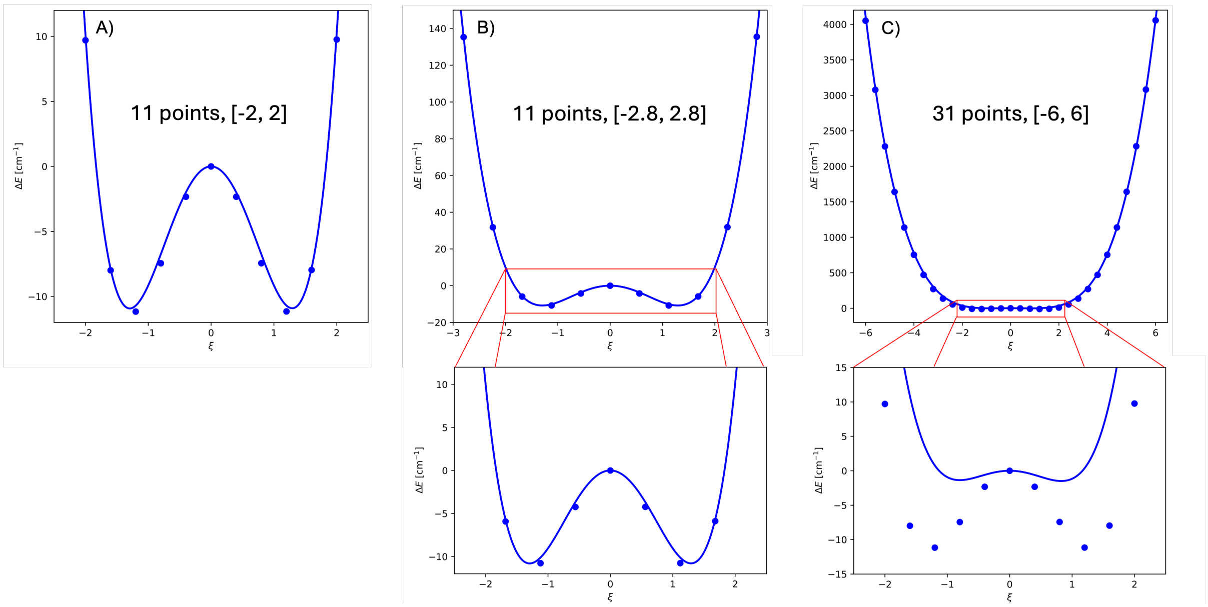

Figure S2: Same as in Figure S1 for different explored ranges.

1.3 Convergence of the Power Expansion of the Potential

To evaluate the quality of the convergence of the power series expansion of the potential, we performed polynomial fittings of the energy computed at 11 nuclear configurations within the range [-2.8, 2.8]. Tables S4 and S5 present energy derivatives and energy levels as a function of the degree G of the fitting polynomial, respectively.

Table S4: Energy derivatives as a function of the degree G of the fitting polynomial.

G	ω	$\partial^3 V/\partial Q^3$	$\partial^4 V/\partial Q^4$	$\partial^5 V/\partial Q^5$	$\partial^6 V/\partial Q^6$
4	$i25.81$	0.01	92.54		
5	$i25.81$	-0.01	92.54	0.04	
6	$i25.99$	-0.01	93.30	0.04	-1.94

Table S5: First seven energy levels as a function of the degree G of the fitting polynomial.

G	E_{ZPE}	E_1	E_2	E_3	E_4	E_5	E_6
4	0.57	14.86	43.92	77.40	115.73	157.89	203.36
5	0.57	14.86	43.92	77.40	115.73	157.89	203.36
6	0.52	14.76	43.76	77.11	115.39	157.71	203.77

2 Wavefunctions of Toy Model Symmetric and Asymmetric DWPs

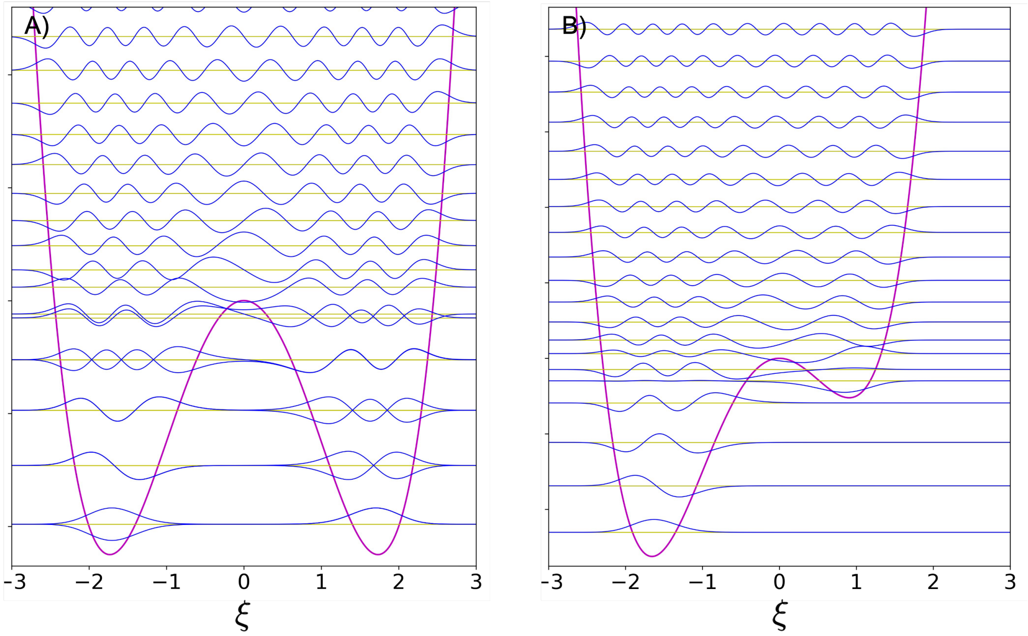

Figure S3: A) The 20 lowest lying anharmonic vibrational energies and wavefunctions of a symmetric DWP with $a = -30$ and $c = 5$. B) Same as in A) but for an asymmetric DWP with $a = -30$, $b = 10$ and $c = 10$.

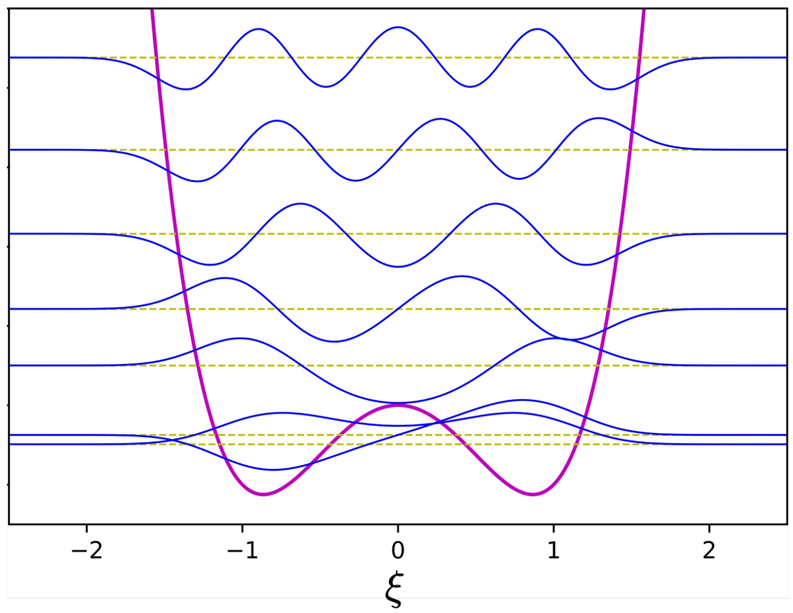

Figure S4: The 7 lowest lying anharmonic vibrational energies and wavefunctions of a symmetric DWP $V = aQ^2 + bQ^3 + cQ^4$ with $a = -30$, $b = 0$, and $c = 20$.

3 Effect of Volume Expansion

The impact of thermal expansion has been considered by applying the same approach to two expanded structures, with volume increases of 1% and 2% with respect to the equilibrium volume V_{eq}. Results are shown in Figure S5.

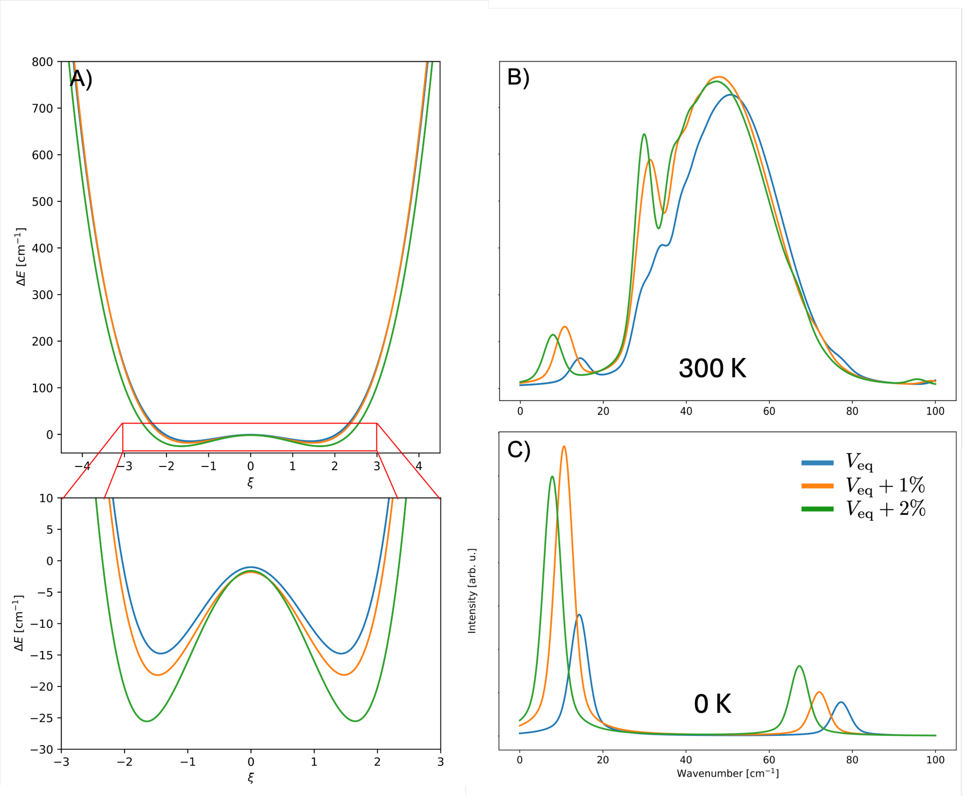

Figure S5: A) DWP of the Pnma phase of thiourea in the range [-4, 4] with 21 energy points. B) and C) Infrared spectrum of the Pnma phase in the 0-100 cm^{-1} spectral range at 300 K and 0 K, respectively. Equilibrium and expanded volumes are color coded: blue for V_{eq}, orange for $V_{eq} + 1\%$ and green for $V_{eq} + 2\%$.
